# Molecular cytogenetic characterization of a new wheat-*Thinopyrum intermedium* homoeologous group-6 chromosome disomic substitution line with resistance to leaf rust and stripe rust

**DOI:** 10.3389/fpls.2022.1006281

**Published:** 2022-09-06

**Authors:** Xiaojun Zhang, Jianbo Li, Yudi Ge, Haixia Guan, Guangrong Li, Shuwei Zhang, Xiaolu Wang, Xin Li, Zhijian Chang, Peng Zhang, Juqing Jia, Cheng Liu

**Affiliations:** ^1^College of Agriculture, Shanxi Agricultural University, Taiyuan, Shanxi, China; ^2^Crop Research Institute, Shandong Academy of Agricultural Sciences, Jinan, Shandong, China; ^3^Plant Breeding Institute, School of Life and Environmental Sciences, The University of Sydney, Sydney, NSW, Australia; ^4^School of Life Sciences, Shanxi University, Taiyuan, Shanxi, China; ^5^School of Life Sciences and Technology, University of Electronic Science and Technology of China, Chengdu, Sichuan, China

**Keywords:** *Thinopyrum intermedium*, GISH, FISH, wheat 55K SNP array, disease resistance

## Abstract

*Thinopyrum intermedium* (JJJ^s^J^s^StSt, 2*n* = 6*x* = 42), a member of tertiary gene pool of hexaploid wheat (*Triticum aestivum* L., AABBDD, 2*n* = 6*x* = 42), provides several beneficial genes for wheat improvement. In this study, line CH51 was developed from the BC_1_F_8_ progeny of a partial wheat-*Th. intermedium* amphiploid TAI8335 (2*n* = 56) and wheat cultivar (cv.) Jintai 170. Somatic metaphase chromosome counting showed that CH51 had stable 42 chromosomes. Genomic *in situ* hybridization (GISH) analysis showed that CH51 had 40 wheat chromosomes and two *Th. intermedium* chromosomes involving translocation between J^s^- and St-genome chromosomes. Non-denaturing fluorescence *in situ* hybridization (ND-FISH) analysis revealed that CH51 lacked a pair of wheat chromosome 6B. Wheat 55K SNP array analysis verified that chromosome 6B had the highest percentage of missing SNP loci in both CH51 and Chinese Spring (CS) nullisomic 6B-tetrasomic 6D (CS-N6BT6D) and had the highest percentage of polymorphic SNP loci between CH51 and cv. Jintai 170. We identified that CH51 was a wheat-*Th. intermedium* T6StS.6J^s^L (6B) disomic substitution line. Disease resistance assessment showed that CH51 exhibited high levels of resistance to the prevalent Chinese leaf rust and stripe rust races in the field. Therefore, the newly developed line CH51 can be utilized as a potential germplasm in wheat disease resistance breeding.

## Introduction

Hexaploid wheat (*Triticum aestivum* L., AABBDD, 2*n* = 6*x* = 42) is one of the most essential cereal crops around the world and provides the major food source for 30% of the global population [International Wheat Genome Sequencing Consortium [IWGSC], 2014]. Wheat diseases such as rusts, powdery mildew, and *Fusarium* head blight (FHB), however, have always been major threats to wheat production in almost all the wheat growing countries. Stripe rust, caused by *Puccinia striiformis* Westend. f. sp. *tritici* (*Pst*), may cause losses up to 70% and even higher (Roelfs et al., 1992; Wellings, 2011). Leaf rust, caused by *P. triticina* Eriks (*Pt*), is another devastating foliar disease, and can also cause severe yield reduction (McIntosh et al., 1995). Developing resistant cultivars is regarded as the most economical and effective means to control diseases. Nevertheless, because of a limited number of effective resistance genes in cultivated wheat and constantly evolving new virulent pathotypes capable of overcoming existing resistance genes in the pathogens, there is an urgent requirement to explore and utilize new resistant resources.

*Thinopyrum intermedium* (Host) Barkworth and D.R. Dewey (JJJ^s^J^s^StSt, 2*n* = 6*x* = 42), a perennial wild relative of hexaploid wheat, possesses many resistance genes, such as those that are resistant to stem rust, stripe rust, leaf rust, and powdery mildew pathogens (Li and Wang, 2009). Due to its high crossability with hexaploid wheat, several resistance genes have been incorporated into wheat (Li et al., 2019a). Up to now, a total of 81 leaf rust (Xu et al., 2022) and 83 stripe rust resistance genes (Li et al., 2020) have been officially named in wheat, respectively, but only *Lr38* (Friebe et al., 1993) and *Yr50* (Liu et al., 2013) were reported from *Th. intermedium*. Therefore, it is of great value to explore new *Th. intermedium* genetic resources for broadening its application in wheat biotic resistance breeding.

Substitution lines between wheat and wild relatives are regarded as the optimal bridging materials for transferring beneficial genes from wild species to cultivated wheat (Liu and Wang, 2005). Compared with addition lines, substitution lines are cytogenetically more stable (Li et al., 2019b) and preferable to produce wheat-alien translocation lines by crossing with the high pairing *ph1b* mutant (Zhang et al., 2017). Chang et al. (2010) reported that wheat-*Th. intermedium* partial amphiploid TAI8335 was highly resistant to leaf rust, stem rust, stripe rust, and powdery mildew. Later, a wheat-*Th. intermedium* disomic substitution line CH51 was selected from the BC_1_F_8_ progeny of TAI8335 and common wheat cultivar (cv.) Jintai 170. In this study, we used chromosome counting, genomic *in situ* hybridization (GISH), non-denaturing fluorescence *in situ* hybridization (ND-FISH), wheat 55K SNP array, and disease responses to: (1) identify the chromosome composition of CH51; (2) confirm the homoeologous relationship of *Th. intermedium* chromosomes in CH51; and (3) evaluate the responses of the newly developed line CH51 to stripe rust, leaf rust, FHB, and powdery mildew.

## Materials and methods

### Plant materials

The materials used in this study included common wheat Chinese Spring (CS), Jinchun 5, Jinmai 33, Jintai 170, Mingxian 169, Nanda 2419, Taichung 29, Sumai 3, Alondra’s, CS nullisomic-tetrasomic lines (CS-N6AT6D, CS-N6BT6D, and CS-N6DT6B), *Th. intermedium* (unknown origin), a partial wheat-*Th. intermedium* amphiploid TAI8335 (2*n* = 8*x* = 56), and its derived line CH51. TAI8335 was developed from BC_1_F_8_ progenies of the cross of Jinchun 5/*Th. intermedium*//Jinmai 33 (Chang et al., 2010). CH51 was selected from BC_1_F_8_ progenies of the cross of Jintai 170/TAI8335//Jintai 170. CS and Taichung 29 were kindly provided by Dr. Zujun Yang, University of Electronic Science and Technology of China, Chengdu, Sichuan, China. Sumai 3 and Alondra’s were kindly provided by Dr. Xiue Wang, Nanjing Agricultural University, Nanjing, Jiangsu, China. All materials are maintained at Shanxi Province Key Laboratory of Crop Genetics and Gene Improvement, College of Agronomy, Shanxi Agricultural University, Taiyuan, Shanxi, China.

### Genomic *in situ* hybridization analysis

Mitotic metaphase chromosomes of CH51 were analyzed by GISH according to the protocols in Zhang et al. (2001). Mitotic metaphase chromosomes were obtained from root tips and were spread according to the procedures in Lang et al. (2018). Total genomic DNA from *Pseudorogneria spicata* was used as a probe and labeled with fluorescein-12-dUTP (yellow-green fluorescence) (Enzo Life Sciences Inc., Farmingdale, NY, United States) using nick translation method. Sheared genomic DNA from CS was used as blocking DNA. Chromosomes were counterstained with propidium iodide (PI), and fluoresced red. GISH images were captured with an epifluorescence Zeiss Axioplan 2 microscope equipped with a SPOT 2.1 CCD camera (Diagnostic Instruments, Sterling Heights, MI, United States).

### Non-denaturing fluorescence *in situ* hybridization analysis

Mitotic metaphase chromosomes of CH51 were further analyzed by ND-FISH according to the procedure of Fu et al. (2015). The oligonucleotide probes Oligo-pSc119.2 and Oligo-pTa535 were used to identify wheat chromosomes according to the description by Tang et al. (2014). Probe Oligo-pSc119.2 was 5’-end labeled with 6-carboxyfluorescein (6-FAM) generating green signals, and probe Oligo-pTa535 were labeled with 6-carboxytetramethylrhodamine (TAMRA) generating red signals (Shanghai Invitrogen Biotechnology Co., Ltd., Shanghai, China). Chromosomes were counterstained with 4’,6-diamidino-2-phenylindole (DAPI) in Vectashield mounting medium (Vector Laboratories, Burlingame, CA, United States). FISH images were captured with an Olympus BX-51 microscope equipped with a DP-70 CCD camera (Shinjuku, Tokyo, Japan).

### Wheat 55K SNP array analysis

Total genomic DNA of CH51, Jintai 170, CS-N6AT6D, CS-N6BT6D, and CS-N6DT6B were extracted using the CTAB method (Chen et al., 2004), and were genotyped on the wheat 55K SNP genotyping arrays (China Golden Marker Biotechnology Company, Beijing, China). There are 53,007 microchip probes per chip, including many diploid markers. Based on CS reference genome sequence IWGSC_RefSeq_v1.0,^1^ a total of 49,060 SNP marker loci had precise physical location information, evenly covering the entire wheat genome. Percentages of the same, polymorphic, or missing SNP loci in each chromosome in CH51, Jintai 170, CS-N6AT6D, CS-N6BT6D, and CS-N6DT6B were obtained by calculating the rate of the same, polymorphic, or missing SNP genotype loci number in total number of SNP loci. Microsoft Excel 2019 (Microsoft, Redmond, WA, United States) was used for data analysis and graphing.

### Disease response evaluation

During the two wheat-growing seasons in 2018–2020, all materials were sown in a randomized complete block design with three replicates for evaluating their responses to stripe rust, leaf rust, powdery mildew, and *Fusarium* head blight (FHB) at the heading stage. Fifteen seeds of each line were sown in 1.5 m rows, spaced 0.25 m apart. Stripe rust was tested at Xindu Experiment Station, Sichuan Academy of Sciences, Chengdu, Sichuan, China. Leaf rust, powdery mildew, and FHB were tested at the Experimental Farm of Shanxi Agricultural University, Jinzhong, Shanxi, China.

Stripe rust responses of Jinchun 5, Jinmai 33, Jintai 170, *Th. intermedium*, TAI8335, CH51, and Taichung 29 were inoculated with a mixture of *Pst* races CYR32, CYR33, and CYR34 (1:1:1 ratio) provided by the Institute of Plant Protection, Gansu Academy of Agricultural Sciences, Lanzhou, Gansu, China. Artificial inoculations were carried out by dusting spores onto the leaves. Wheat cv. Taichung 29 was used as the susceptible control. When spores were fully developed on Taichung 29, infection types (ITs) were recorded based on a 0–4 scale, where 0, 0; 1, 2, 3, and 4 indicated immune, highly resistant, resistant, moderately resistant, moderately susceptible, and susceptible, respectively (McIntosh et al., 1995).

Leaf rust reactions of all tested materials were recorded after being inoculated with a mixture of prevalent *Pt* races TRT, TRJ, and KHJ (1:1:1 ratio), which were collected from wheat-growing areas in northern China (Sheng et al., 2022). Inoculation method was according to Sheng et al. (2022). Wheat cv. Nanda 2419 was used as the susceptible control. ITs were recorded as 0–4 scale according to McIntosh et al. (1995).

Powdery mildew responses of all tested materials were evaluated after being inoculated with *Blumeria graminis* f. sp. *tritici* (*Bgt*) race E09 provided by the Institute of Plant Protection, Chinese Academy of Agricultural Sciences, Beijing, China. Inoculations were carried out as described by Xiang et al. (1994). When conidia were spread across the susceptible control Mingxian 169, ITs were recorded on a 0–9 scale, where 0, 0; 1–2, 3–4, 5–6, and 7–9 indicated immune, near immune, highly resistant, moderately resistant, moderately susceptible, and highly susceptible, respectively (Sheng and Duan, 1991).

*Fusarium* head blight (FHB) reactions of all tested materials were recorded after being inoculated with *Fusarium* pathotype F0609 provided by Dr. Xiue Wang, Nanjing Agricultural University, Nanjing, Jiangsu, China. Plants were inoculated as described by Bai et al. (1999) and Zhang et al. (2020) when a spike was just beginning to flower. Sumai 3 was used as the resistant control, and Alondra’s was used as the susceptible control. Disease severity was recorded 27 days post inoculation according to a 0–4 scale, where 0, 1, 2, 3, and 4 indicated immune, resistant, moderately resistant, moderately susceptible, and susceptible, respectively (Zhang et al., 2020).

## Results

### Cytological characterization of CH51 using genomic *in situ* hybridization and fluorescence *in situ* hybridization analyses

Wheat-*Th. intermedium* derived line CH51 was selected from the BC_1_F_8_ progeny of the cross of Jintai 170/TAI8335//Jintai 170. A total of 30 CH51 seeds were germinated for chromosome counting. The result showed that the somatic metaphase chromosome number of all 30 seeds are 2*n* = 42, confirming its cytogenetic stability.

**FIGURE 1:**
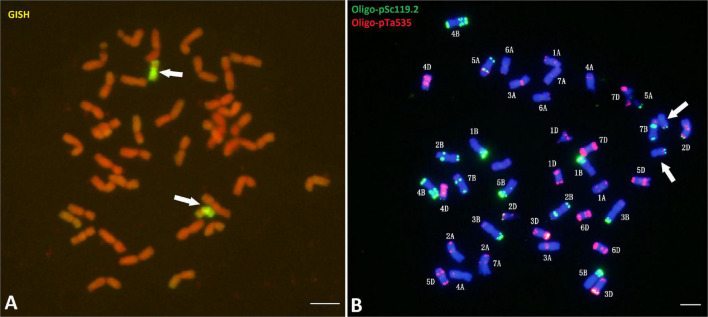
Genomic *in situ* hybridization (GISH) and Non-denaturing fluorescence *in situ* hybridization (ND-FISH) analyses of mitotic metaphase chromosomes of CH51. **(A)** *Pseudorogneria spicata* total genomic DNA was used as a probe (yellow-green) in GISH analysis. Chromosomes were counterstained with propidium iodide (PI) and fluoresced red. **(B)** Probes Oligo-pSc119.2-1 (green) and Oligo-pTa535-1 (red) were used in ND-FISH analysis. Chromosomes were counterstained with 4’,6-diamidino-2-phenylindole (DAPI) and fluoresced blue. Arrows **(A, B)** point to the alien translocation chromosomes. Bars, 10 μm.

Genomic *in situ* hybridization (GISH) analysis using *Ps. spicata* genomic DNA as a probe showed that CH51 had 40 wheat chromosomes and two *Th. intermedium* chromosomes displaying stronger hybridization signals along the entire short arm and at the telomeric region of the long arm (Figure 1A). According to Chen et al. (1999), GISH using the diploid progenitor *Ps. spicata* as a probe could label the entire length of St-genome chromosomes, the pericentromeric and telomeric regions of J^s^-genome chromosomes, and the telomeres of J-genome chromosomes, indicating that the *Thinopyrum* chromosome in CH51 involved translocation between J^s^- and St-genome chromosomes. Sequential ND-FISH with probes Oligo-pSc119.2 and Oligo-pTa535 revealed that CH51 had 40 wheat chromosomes and a pair of unknown chromosomes, which substitute for the wheat chromosome 6B (Figure 1B). Therefore, we concluded that CH51 is a wheat-*Th. intermedium* T?StS.?J^s^L (6B) disomic substitution line.

### Wheat 55K SNP array analysis

**FIGURE 2:**
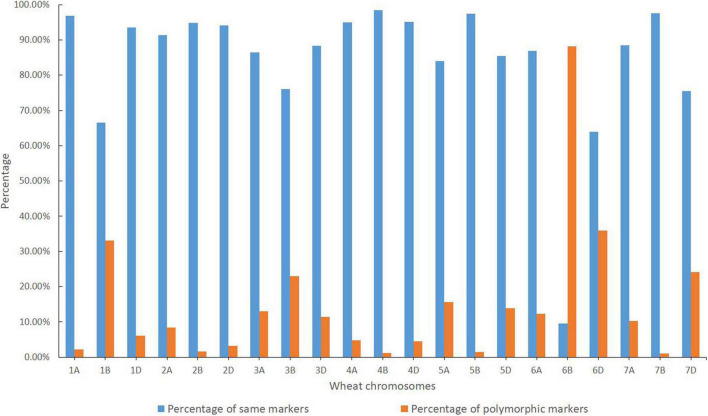
Wheat 55K SNP array analysis. Orange and blue colors indicate the percentages of the same and polymorphic SNP loci in each chromosome in the total number of SNP loci between CH51 and wheat parent Jintai 170, respectively.

Based on the reference genome sequence of CS, a total of 49,060 SNP loci having precise physical location information were used in the wheat SNP array analysis. Among them, a total of 46,380 and 48,288 valid SNP loci were identified in CH51 and Jintai 170, respectively (Table 1). A total of 41,186 SNP loci were common between CH51 and Jintai 170. As shown in Figure 2, chromosome 6B shared the minimum percentage of the same SNP loci (9.58%) between CH51 and Jintai 170, whereas other chromosomes shared much higher percentages of the same SNP loci ranging from 63.89% (on 6D) to 98.40% (on 4B). A total of 7,403 SNP loci were polymorphic between CH51 and Jintai 170. As shown in Figure 2, chromosome 6B had the highest percentage of polymorphic SNP loci (88.18%) between CH51 and Jintai 170, whereas other chromosomes had lower percentages of polymorphic SNP loci ranging from 1.05% (on 7B) to 35.94% (on 6D). In addition, a total of 471 SNP loci (0.96%) were simultaneously missing in both CH51 and Jintai 170, which could not be used in the statistical analysis (Table 1). The result indicated that wheat chromosome 6B in CH51 was substituted by a pair of homoeologous group-6 chromosome from *Th. intermedium*.

**TABLE 1:** SNP genotyping data obtained using wheat 55K SNP arrays for CH51 and wheat parent Jintai 170.

				CH51 vs. Jintai 170					
Chromosome	No. of markers	No. of valid markers in CH51	No. of valid markers in Jintai 170	No. of same markers	Percentage of same markers	No. of polymorphic markers	Percentage of polymorphic markers	No. of simultaneous missing markers	Percentage of simultaneous missing markers
1A	2625	2581	2587	2543	96.88%	57	2.17%	25	0.95%
1B	2595	2556	2562	1728	66.59%	859	33.10%	8	0.31%
1D	2138	2107	2108	1999	93.50%	129	6.03%	10	0.47%
2A	2622	2585	2599	2394	91.30%	219	8.35%	9	0.35%
2B	2600	2486	2499	2467	94.88%	42	1.62%	91	3.50%
2D	2247	2173	2177	2115	94.13%	72	3.20%	60	2.67%
3A	2174	2130	2140	1878	86.38%	284	13.07%	12	0.55%
3B	2595	2547	2559	1974	76.07%	598	23.04%	23	0.89%
3D	1693	1600	1679	1495	88.30%	194	11.46%	4	0.24%
4A	2592	2569	2567	2460	94.91%	123	4.75%	9	0.34%
4B	2556	2534	2541	2515	98.40%	32	1.25%	9	0.35%
4D	1420	1403	1410	1350	95.07%	65	4.58%	5	0.35%
5A	2611	2580	2587	2193	83.99%	409	15.66%	9	0.35%
5B	2586	2543	2548	2519	97.41%	38	1.47%	29	1.12%
5D	1737	1716	1717	1483	85.38%	242	13.93%	12	0.69%
6A	2623	2519	2573	2279	86.89%	324	12.35%	20	0.76%
6B	2547	947	2478	244	9.58%	2246	88.18%	57	2.24%
6D	1728	1681	1690	1104	63.89%	621	35.94%	3	0.17%
7A	2579	2530	2533	2280	88.41%	266	10.31%	33	1.28%
7B	2487	2444	2441	2425	97.51%	26	1.05%	36	1.44%
7D	2305	2149	2293	1741	75.53%	557	24.16%	7	0.31%
Total	49060	46380	48288	41186	83.95%	7403	15.09%	471	0.96%

To verify whether wheat chromosome 6B was absent in CH51, CS-N6BT6D, CS-N6AT6D, and CS-N6DT6B were also included in genotyping with wheat 55K SNP genotyping arrays. As shown in Supplementary Table 1, chromosome 6B in CH51 had the highest percentage of missing SNP loci of 62.82%, whereas other chromosomes had much lower percentages, ranging from 0.86% (4B) to 6.77% (6D). For CS-N6BT6D, because it lacks the wheat chromosome 6B, we speculated that it should have a highest percentage of missing SNP loci on chromosome 6B, which was confirmed by SNP array analysis that chromosome 6B in CS-N6BT6D had the highest percentage (66.98%) of missing SNP loci (Supplementary Table 1). Combined with FISH-GISH results, it was demonstrated that CH51 was a wheat-*Th. intermedium* T6StS.6J^s^L (6B) disomic substitution line.

### Assessment of responses to leaf rust, stripe rust, powdery mildew, and *Fusarium* head blight

**FIGURE 3:**
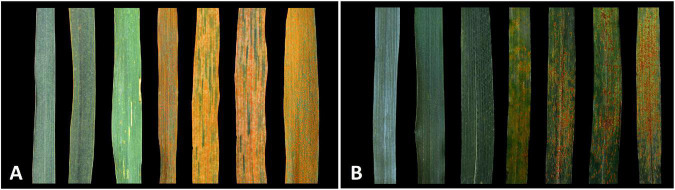
Stripe rust and leaf rust responses of tested materials at the heading stage. **(A)** A mixture of stripe rust races CYR32, CYR33, and CYR34 (1:1:1 ratio) were inoculated on (from left to right): *Thinopyrum intermedium*, TAI8335, CH51, Jinchun 5, Jinmai 33, Jintai 170, Taichung 29. **(B)** A mixture of leaf rust races TRT, TRJ, and KHJ (1:1:1 ratio) were inoculated on (from left to right): *Th. intermedium*, TAI8335, CH51, Jinchun 5, Jinmai 33, Jintai 170, Nanda 2419.

At the heading stage, responses to stripe rust, leaf rust, powdery mildew, and FHB were recorded in Table 2. For stripe rust (Figure 3A) and leaf rust (Figure 3B), the susceptible control Taichung 29 and Nanda 2419, wheat parents Jinchun 5, Jinmai 33 and Jintai 170 were susceptible (IT 3+ or 4), whereas *Th. intermedium* and TAI8335 were immune or highly resistant (IT 0 or 0;), and CH51 was highly resistant or resistant (IT ;1=). For powdery mildew (Supplementary Figure 1A), the susceptible control Mingxian 169, wheat parents Jinchun 5, Jinmai 33 and Jintai 170, and CH51 were highly susceptible (IT 9), whereas *Th. intermedium* and TAI8335 were immune (IT 0). For FHB (Supplementary Figure 1B), the susceptible control Alondra’s, wheat parents Jinchun 5, Jinmai 33 and Jintai 170, and CH51 were highly susceptible (IT 3+ or 4), whereas TAI8335 was moderately resistant (IT 2), and the resistant control Sumai 3 was highly resistant (IT 1). Therefore, we concluded that the translocation chromosome T6StS.6J^s^L in CH51 might carry genes for resistance to stripe rust and leaf rust in the field, but not resistance genes to powdery mildew and FHB.

**TABLE 2:** Responses of tested materials to stripe rust (*Pst*), leaf rust (*Pt*), powdery mildew (*Bgt*), and FHB at the heading stage.

Materials	*Pst*	*Pt*	*Bgt*	FHB
	CYR32 + CYR33 + CYR34	TRT + TRJ + KHJ	E09	F0609
*Thinopyrum intermedium*	0	0	0	–
TAI8335	0;	0	0	2
CH51	;1	;1=	9	3+
Jinchun 5	4	3+	9	4
Jinmai 33	4	4	9	4
Jintai 170	4	4	9	4
Mingxian 169	–	–	9	–
Nanda 2419	–	4	–	–
Taichung 29	4	–	–	–
Sumai 3	–	–	–	1
Alondra’s	–	–	–	4

“–”, not tested.

## Discussion

The homoeologous group-6 chromosomes of wild relatives of common wheat carry many desirable genes, such as higher micronutrient contents in grain and resistance to stripe rust, leaf rust, and powdery mildew. For example, Ardalani et al. (2016) reported that wheat-*Th. bessarabicum* substitution line DS6E^b^ (6D) and translocation line T6E^b^S.6DL had higher iron and zinc contents than the recipient wheat cv. “Roushan” and demonstrated that the gene(s) conferring high Fe and Zn contents was located on the short arm of *Th. bessarabicum* chromosome 6E^b^. Song et al. (2016) revealed that the bin of fraction length (FL) 0.81–1.00 of the long arm of *Agropyron cristatum* chromosome 6P carried leaf rust resistance gene(s). The powdery mildew resistance gene *Pm21* derived from *Haynaldia villosa* is located on 6VS and encodes a CC-NBS-LRR (NLR) protein (He et al., 2018; Xing et al., 2018). Li et al. (2020) mapped a new stripe rust resistance gene *Yr83* to the bin of FL 0.73–1.00 of the long arm of *Secale cereale* chromosome 6R. Recently, Zhang et al. (2021) isolated stem rust resistance genes *Sr26* and *Sr61* from *Th. ponticum* chromosomes 6Ae#1 and 6Ae#3, respectively, which encode unrelated *NLR* genes and remain effective against all known *Pgt* races, including the widely virulent *Pgt* race Ug99 (TTKSK). In the present study, we identified a wheat-*Th. intermedium* T6StS.6J^s^L (6B) disomic substitution line CH51, which exhibited high levels of resistance to the prevalent Chinese leaf rust and stripe rust races in the field (Figure 3).

After transferring alien chromosomes into wheat, it is important to efficiently track alien chromosome(s) in wheat-alien introgression lines. GISH is regarded as a powerful and reliable technique for determining the genomic origin, size of introgressed fragments and breakpoint positions of the introgressions (Li et al., 2020). In this study, we used GISH analysis with *Ps. spicata* genomic DNA as a probe and showed that CH51 carried a pair of *Th. intermedium* J^s^-/St-genome translocation chromosomes (Figure 1A). In addition, FISH is an efficient tool for the identification of wheat and alien chromosomes in wheat-alien introgression lines (Tang et al., 2014). We used FISH analysis to show that CH51 lacked a pair of wheat chromosome 6B but had a pair of *Th. intermedium* chromosomes (Figure 1B). A combination of GISH and FISH indicated that CH51 is a wheat-*Th. intermedium* T6StS.6J^s^L (6B) disomic substitution line.

With the rapid development of sequencing technologies, SNP array analysis is becoming increasingly popular in high-throughput genotyping wheat and wild relatives because of its high-density loci and reasonable cost (Winfield et al., 2016). Recently, SNP arrays also play a vital role in detecting the homoeologous relationships between wheat and alien chromosomes in wheat-alien introgression lines (Li et al., 2019b; Wang et al., 2020, 2022). In this study, results from the wheat 55K SNP array showed that chromosome 6B had the highest percentage of polymorphic SNP loci between CH51 and wheat parent Jintai 170 (Figure 2 and Table 1) and also had the highest percentage (62.82%) of missing SNP loci in CH51 (Supplementary Table 1). Combining with the cytology result, we concluded that CH51 is a wheat-*Th. intermedium* T6StS.6J^s^L (6B) disomic substitution line. In addition, SNP array results also verified that the tested materials, CS-N6BT6D, CS-N6AT6D, and CS-N6DT6B, used in the current study are correct, which correspond to the highest percentage of missing SNP loci of 62.82% (6B), 67.82% (6A), 76.50% (6D), respectively (Supplementary Table 1).

In this study, TAI8335 exhibited high levels of resistance to stripe rust, leaf rust, powdery mildew, and FHB in the field. Our results showed that the translocation chromosome T6StS.6J^s^L in CH51 carried resistance genes for stripe rust and leaf rust (Figure 3), but not for powdery mildew and FHB (Supplementary Figure 1). Therefore, the other six *Th. intermedium* chromosomes in TAI8335 should carry powdery mildew and FHB resistance genes and might also carry additional stripe rust and leaf rust resistance genes. For the future research, we will (1) backcross CH51 with the high pairing CS *ph1b* mutant to develop small segmental 6StS or 6J^s^L translocation lines for reducing the potential linkage drag and mapping the two genes; and (2) backcross TAI8335 with common wheat for transferring powdery mildew and FHB resistance genes and/or other stripe rust and leaf rust resistance genes.

## Conclusion

A wheat-*Th. intermedium* T6StS.6J^s^L (6B) disomic substitution line CH51 was developed from the BC_1_F_8_ progeny of a partial wheat-*Th. intermedium* amphiploid TAI8335 and common wheat cv. Jintai 170. The chromosome composition of CH51 is 14A + 12B + 14D + 2T6StS.6J^s^L. CH51 exhibited high levels of resistance to the prevalent Chinese leaf rust and stripe rust races in the field. Therefore, the newly developed line CH51 can be utilized as a potential germplasm in wheat disease resistance breeding.

## Data availability statement

The datasets presented in this study can be found in online repositories. The names of the repository/repositories and accession number(s) can be found in the article/Supplementary material.

## Author contributions

CL, JJ, and XZ conceived and designed the research. ZC and XZ contributed to the development of the materials. ZC performed the GISH experiment. GL performed the FISH experiment. HG, JL, and JJ carried out wheat 55K SNP analysis. ZC, XZ, and YG performed powdery mildew and FHB tests. SZ, XL, and JJ performed leaf rust test. CL and XW performed stripe rust test. JL wrote the manuscript. PZ, ZC, XZ, JJ, and CL helped with analysis and edited the manuscript. All authors contributed to the manuscript and approved the submitted version.

## Footnotes

1
http://wheat-urgi.versailles.inra.fr/Seq-Repository/

## Supplementary material

Supplementary Figure 1 Powdery mildew and *Fusarium* head blight (FHB) responses of tested materials at the heading stage. **(A)** Powdery mildew race E09 was inoculated on (from left to right): *Th. intermedium*, TAI8335, CH51, Jinchun 5, Jinmai 33, Jintai 170, Mingxian 169. **(B)** *Fusarium* pathogen F0609 was inoculated on (from left to right): Sumai 3, Jinchun 5, Jinmai 33, TAI8335, Jintai 170, CH51, Alondra’s.

## References

[B1] Ardalani S. Mirzaghaderi G. Badakhshan H. (2016). A Robertsonian translocation from *Thinopyrum bessarabicum* into bread wheat confers high iron and zinc contents. *Plant Breed.* 135 286–290. 10.1111/pbr.12359

[B2] Bai G. Kolb F. L. Shaner G. Domier L. L. (1999). Amplified fragment length polymorphism markers linked to a major quantitative trait locus controlling scab resistance in wheat. *Phytopathology* 89 343–348. 10.1094/PHYTO.1999.89.4.343 18944781

[B3] Chang Z. Zhang X. Yang Z. Zhan H. Li X. Liu C. (2010). Characterization of a partial wheat-*Thinopyrum intermedium* amphiploid and its reaction to fungal diseases of wheat. *Hereditas* 147 304–312. 21166800 10.1111/j.1601-5223.2010.02156.x

[B4] Chen K. Li F. Xu C. Zhang S. Fu C. (2004). An efficient macro-method of genomic DNA isolation from *Actinidia chinensis* leaves. *Hereditas* 26 529–531. 15640056

[B5] Chen Q. Conner R. L. Laroche A. Fedak G. Thomas J. B. (1999). Genomic origins of *Thinopyrum* chromosomes specifying resistance to wheat streak mosaic virus and its vector, *Aceria tosichella*. *Genome* 42 289–295. 10.1139/g98-131

[B6] Friebe B. Jiang J. Gill B. S. Dyck P. L. (1993). Radiation-induced nonhomoeologous wheat-*Agropyron intermedium* chromosomal translocations conferring resistance to leaf rust. *Theor. Appl. Genet.* 86 141–149. 10.1007/BF00222072 24193453

[B7] Fu S. Chen L. Wang Y. Li M. Yang Z. Qiu L. (2015). Oligonucleotide probes for ND-FISH analysis to identify rye and wheat chromosomes. *Sci. Rep.* 5:10552. 10.1038/srep10552 25994088 PMC4440213

[B8] He H. Zhu S. Zhao R. Jiang Z. Ji Y. Ji J. (2018). *Pm21*, encoding a typical CC-NBS-LRR protein, confers broad-spectrum resistance to wheat powdery mildew disease. *Mol. Plant* 11 879–882. 10.1016/j.molp.2018.03.004 29567454

[B9] International Wheat Genome Sequencing Consortium [IWGSC] (2014). A chromosome-based draft sequence of the hexaploid bread wheat (*Triticum aestivum*) genome. *Science* 345:1251788. 10.1126/science.1251788 25035500

[B10] Lang T. La S. Li B. Yu Z. Chen Q. Li J. (2018). Precise identification of wheat-*Thinopyrum intermedium* translocation chromosomes carrying resistance to wheat stripe rust in line Z4 and its derived progenies. *Genome* 61 177–185. 10.1139/gen-2017-0229 29470932

[B11] Li H. Wang X. (2009). *Thinopyrum ponticum* and *Th. intermedium*: The promising source of resistance of fungal and viral diseases of wheat. *J. Genet. Genomics* 36 557–565. 10.1016/S1673-8527(08)60147-2 19782957

[B12] Li J. Chen Q. Zhang P. Lang T. Hoxha S. Li G. (2019a). Comparative FISH and molecular identification of new stripe rust resistant wheat-*Thinopyrum intermedium* ssp. *trichophorum* introgression lines. *Crop J.* 7 819–829. 10.1016/j.cj.2019.06.001

[B13] Li J. Yao X. Yang Z. Cheng X. Yuan F. Liu Y. (2019b). Molecular cytogenetic characterization of a novel wheat-*Psathyrostachys huashanica* Keng 5Ns (5D) disomic substitution line with stripe rust resistance. *Mol. Breed.* 39:109. 10.1007/s11032-019-1014-3

[B14] Li J. Dundas I. Dong C. Li G. Trethowan R. Yang Z. (2020). Identification and characterization of a new stripe rust resistance gene *Yr83* on rye chromosome 6R in wheat. *Theor. Appl. Genet.* 133 1095–1107. 10.1007/s00122-020-03534-y 31955232

[B15] Liu J. Chang Z. Zhang X. Yang Z. Li X. Jia J. (2013). Putative *Thinopyrum intermedium*-derived stripe rust resistance gene *Yr50* maps on wheat chromosome arm 4BL. *Theor. Appl. Genet.* 126 265–274. 10.1007/s00122-012-1979-3 23052018

[B16] Liu S. Wang H. (2005). Characterization of a wheat-*Thinopyrum intermedium* substitution line with resistance to powdery mildew. *Euphytica* 143 229–233. 10.1007/s10681-005-3862-7

[B17] McIntosh R. A. Wellings C. R. Park R. F. (1995). *Wheat rusts: An Atlas of resistance genes.* Melbourne: CSIRO Publishing.

[B18] Roelfs A. P. Singh R. P. Saari E. E. (1992). *Rust diseases of wheat: Concepts and methods of disease management.* Mexico: CIMMYT.

[B19] Sheng B. Q. Duan X. Y. (1991). Modification on the evaluation methods of 0-9 level of powdery mildew infection on wheat. *Biotech. J. Agric. Sci.* 9 37–39.

[B20] Sheng D. Liu M. Zhang X. Qiao L. Chang L. Guo H. (2022). Characterization of leaf rust resistance in a set of wheat-*Thinopyrum* amphiploid-derived hexaploid breeding lines. *Crop Prot.* 156:105956. 10.1016/j.cropro.2022.105956

[B21] Song L. Lu Y. Zhang J. Pan C. Yang X. Li X. (2016). Physical mapping of *Agropyron cristatum* chromosome 6P using deletion lines in common wheat background. *Theor. Appl. Genet.* 129 1023–1034. 10.1007/s00122-016-2680-8 26920547

[B22] Tang Z. Yang Z. Fu S. (2014). Oligonucleotides replacing the roles of repetitive sequences pAs1, pSc119.2, pTa-535, pTa71, CCS1, and pAWRC.1 for FISH analysis. *J. Appl. Genet.* 55 313–318. 10.1007/s13353-014-0215-z 24782110

[B23] Wang S. Wang C. Feng X. Zhao J. Deng P. Wang Y. (2022). Molecular cytogenetics and development of St-chromosome-specific molecular markers of novel stripe rust resistant wheat-*Thinopyrum intermedium* and wheat-*Thinopyrum ponticum* substitution lines. *BMC Plant Biol.* 22:111. 10.1186/s12870-022-03496-x 35279089 PMC8917741

[B24] Wang Y. Cao Q. Zhang J. Wang S. Chen C. Wang C. (2020). Cytogenetic analysis and molecular marker development for a new wheat-*Thinopyrum ponticum* 1J^s^ (1D) disomic substitution line with resistance to stripe rust and powdery mildew. *Front. Plant Sci.* 11:1282. 10.3389/fpls.2020.01282 32973841 PMC7472378

[B25] Wellings C. R. (2011). Global status of stripe rust: A review of historical and current threats. *Euphytica* 179 129–141. 10.1007/s10681-011-0360-y

[B26] Winfield M. O. Allen A. M. Burridge A. J. Barker G. L. Benbow H. R. Wilkinson P. A. (2016). High-density SNP genotyping array for hexaploid wheat and its secondary and tertiary gene pool. *Plant Biotechnol. J.* 14 1195–1206. 10.1111/pbi.12485 26466852 PMC4950041

[B27] Xiang Q. J. Sheng B. Q. Zhou Y. L. Duan X. Y. Zhang K. C. (1994). Analyses of resistance genes of three differential varieties to the isolates of *Blumeria graminis* f. sp. *tritic*i in wheat. *Acta Agric. Boreali Sin.* 9 94–97. 10.3321/j.issn:1000-7091.1994.02.018 30704229

[B28] Xing L. Hu P. Liu J. Witek K. Zhou S. Xu J. (2018). *Pm21* from *Haynaldia villosa* encodes a CC-NBS-LRR protein conferring powdery mildew resistance in wheat. *Mol. Plant* 11 874–878. 10.1016/j.molp.2018.02.013 29567451

[B29] Xu X. Kolmer J. Li G. Tan C. Carver B. F. Bian R. (2022). Identification and characterization of the novel leaf rust resistance gene *Lr81* in wheat. *Theor. Appl. Genet.* 135 2725–2734. 10.1007/s00122-022-04145-5 35716201

[B30] Zhang J. Hewitt T. C. Boshoff W. H. P. Dundas I. Upadhyaya N. Li J. (2021). A recombined *Sr26* and *Sr61* disease resistance gene stack in wheat encodes unrelated *NLR* genes. *Nat. Commun*. 12:3378. 10.1038/s41467-021-23738-0 34099713 PMC8184838

[B31] Zhang P. Dundas I. S. Xu S. S. Friebe B. McIntosh R. A. Raupp W. J. (2017). Chromosome engineering techniques for targeted introgression of rust resistance from wild wheat relatives. *Methods Mol. Biolol.* 1659 163–172. 10.1007/978-1-4939-7249-4_14 28856649

[B32] Zhang P. Friebe B. Lukaszewski A. J. Gill B. S. (2001). The centromere structure in Robertsonian wheat-rye translocation chromosomes indicates that centric breakage-fusion can occur at different positions within the primary constriction. *Chromosoma* 110 335–344. 10.1007/s004120100159 11685533

[B33] Zhang X. J. Xiao J. Wang H. Y. Qiao L. Y. Li X. Guo H. J. (2020). Evaluation of resistance to *Fusarium* head blight in *Thinopyrum*-derived wheat lines. *Acta Agron. Sin.* 46 62–73. 10.3724/SP.J.1006.2020.91015

