# Supplementary figures and images for: Molecular cytogenetic characterization of a new wheat-Thinopyrum intermedium homoeologous group-6 chromosome disomic substitution line with resistance to leaf rust and stripe rust
Source: Front Plant Sci. 2022 Sep 6;13:1006281. doi: 10.3389/fpls.2022.1006281 (PMC9486089; doi:10.3389/fpls.2022.1006281)

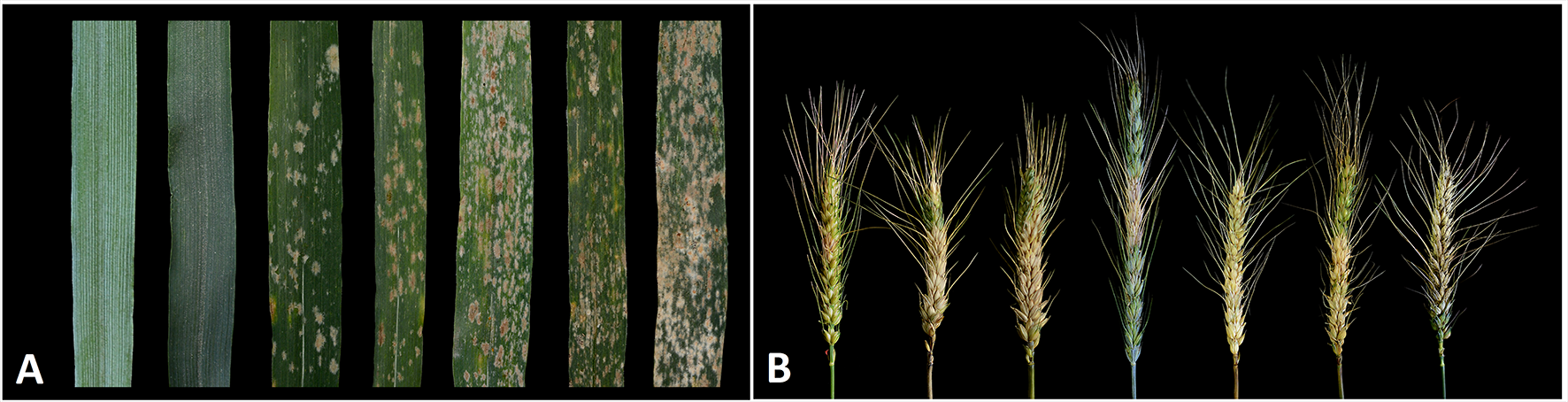

Supplement: Supplementary Figure 1 — Powdery mildew and Fusarium head blight (FHB) responses of tested materials at the heading stage. (A) Powdery mildew race E09 was inoculated on (from left to right): Th. intermedium, TAI8335, CH51, Jinchun 5, Jinmai 33, Jintai 170, Mingxian 169. (B) Fusarium pathogen F0609 was inoculated on (from left to right): Sumai 3, Jinchun 5, Jinmai 33, TAI8335, Jintai 170, CH51, Alondra’s. [file Image_1.TIF]
